# Long-term follow-up on recovery, return to use and sporting activity: a retrospective study of 236 operated colic horses in Finland (2006–2012)

**DOI:** 10.1186/s13028-016-0273-9

**Published:** 2017-01-05

**Authors:** Isa Anna Maria Immonen, Ninja Karikoski, Anna Mykkänen, Tytti Niemelä, Jouni Junnila, Riitta-Mari Tulamo

**Affiliations:** 1 Department of Equine and Small Animal Medicine, Faculty of Veterinary Medicine, University of Helsinki, 00014 Helsinki, Finland; 2 4 Pharma, 20520 Turku, Finland

**Keywords:** Colic, Surgery, Complication, Hernia, Survival, Long-term, Return to use, Performance, Retrospective, Owner satisfaction

## Abstract

**Background:**

Surgical treatment of colic is expensive and complications may occur. Information on the prognosis and the use of the horse after surgery for colic is important for surgeons and owners. Current literature on return to athletic function after celiotomy is limited. The present study reviewed surgical cases of the Veterinary Teaching Hospital, Helsinki, Finland for 2006–2012. The aim was to follow the population of horses of different breeds for surgical findings, postsurgical complications, long-term recovery and prognosis. The findings and their influence on survival, return to previous or intended use and performance were assessed.

**Results:**

Most of the operated horses (82.6%; 195/236) recovered from anesthesia and 74.9% (146/195) were discharged. The total follow-up time was 8 years and 10 months and the median survival time 79.2 months. Age of the horse, location of the abdominal lesion (small vs. large intestine), incidence of postoperative colic, surgical site infection, incisional hernia or convalescence time after surgery, did not significantly affect the probability of performing in the previous or intended discipline after the surgery. A majority of the discharged horses (83.7%) was able to perform in the previous or intended discipline and 78.5% regained their former or higher level of performance. Operated horses had 0.18 colic episodes per horse-year during the long-term follow-up. The incidence of colic was 20.0% within the first year after surgery. Horses operated for large intestinal colic were 3.3-fold more prone to suffer postoperative colic than horses operated for small intestinal colic. The majority of the owners (96.3%) were satisfied with the veterinary care and nearly all (98.5%) evaluated the recovery after the colic surgery to be satisfactory or above.

**Conclusions:**

If the horse survives to discharge, prognosis for long-term survival and return to previous level of sporting activity and performance was good after colic surgery in a population of horses of different breeds. None of the factors studied were found to decrease the probability of performing in the same or intended discipline after surgery. The majority of horses were able to return to their previous activity and perform satisfactorily for several years after surgery.

## Background

It is important to assess long-term survival for horses that have been operated on for colic. Location and severity of the lesion, surgical procedures performed, age of the horse, complications and incidence of postoperative colic episodes affect the convalescence time and subsequent performance capacity of the horse. Therefore, obtaining more information and clarifying these factors is helpful to clinicians attempting to prognosticate the long-term outcome for individual horses and for their owners to take the most feasible decision.

Several studies that evaluated the recovery of horses after a colic surgery showed that 66–91% of horses were alive for at least 1 year after the celiotomy [1–8]. Comprehensive studies on long-term survival and return to use and performance are challenging as substantial numbers of the horses and information is often lost to follow-up [7]. The most common long-term complications that affect the return to use and performance are incisional hernia and postoperative colic [2–4]. Horses that developed an incisional hernia, were 7–14 times less likely to return to athletic use [6, 7]. According to previous reports postoperative colic is more common in horses with strangulating small intestinal lesions or right dorsal displacement of the colon and it occurs within 100 days after surgery [1, 9, 10]. The incidence of postoperative colic in long-term follow-up for longer than 1.7 years and the effect on performance has not been documented to our knowledge. Postoperative racing performance and use have been studied mainly in Thoroughbred horses, and 63–76% of the operated horses returned to racing [11–13]. Two recent studies have evaluated the return to previous sporting activity and also to the previous performance level in a population of horses of different breeds. In addition, the horse-owners’ levels of satisfaction were assessed. According to these two reports, owners expressed that 76–86% of the horses to have regained their previous or intended uses [6, 7].

The objectives of this retrospective study were to evaluate the long-term effect of celiotomy in a heterogeneous (for sex and breed) population of horses in Finland and specifically to determine: First, the short-term and long-term survival of horses over 8 years after colic surgery. Second, evaluate the effect that the age of the horse, location of the surgical lesion (small intestine vs. large intestine), postoperative complications (surgical site infection SSI, incisional hernia, colic), has upon postoperative convalescence time and return to athletic function. Third, determine the incidence rate of colic episodes long-term after the colic surgery (small vs. large intestine lesion). Fourth, evaluate owner satisfaction regarding the outcome after colic surgery and incorporate it into this study.

The hypotheses were that a majority of horses discharged from the hospital after colic surgery would return to their intended use and regain their previous level of performance and that the owners would be satisfied with the end results. It was also hypothesised that horses with small intestinal lesions would have longer postoperative convalescence time and shorter long-term survival time compared with the horses with large intestinal lesions. Horses with small intestinal lesions were therefore hypothesised to be more prone to postoperative colic episodes than horses with large intestinal lesions. The final hypothesis was that postoperative colic episodes and incisional complications or incisional hernia would have a negative effect on the return to use and performance.

## Methods

This retrospective study included 236 horses that had undergone ventral midline celiotomy in the Helsinki University Equine Teaching Hospital, Finland during the 2006 to 2012 period. Patients were followed either until their death or until the end of the study (30th November 2014).

Patient records were reviewed for patient data including: age (0–14 years/≥15 years [7]); breed (Standardbred/Warmblood/Finnhorse/other horse breed/pony); gender (mare/stallion/gelding); insurance status (yes/no); location of the lesion (small intestine/large intestine); diagnosis category, surgical findings and treatment procedures (intestinal gas removal yes/no; enterotomy yes/no; resection yes/no); postoperative complications during hospitalization (SSI/incisional dehiscence defined as partial or complete rupture of one or more layers of stitches/incisional hernia/reflux/ileus/continuous pain or colic/peritonitis/laminitis); re-laparotomy (time/cause/surgical findings and treatment); death postoperatively during hospitalization and the date of the discharge were recorded. National competition records were searched for both pre- and postoperative entries in national and international show jumping, dressage, eventing and harness racing to evaluate postoperative performance (Heppa® database of the Finnish Trotting Association Hippos, Helsinki, Finland; Kipa® database of The Equestrian Federation of Finland, Valo, Finland). The short-term survival period was defined as the time from the recovery after anesthesia to the time of discharge. The long-term survival was defined as time from the discharge till the end of the study/death of the horse.

A telephone interview was held with the owners of 125 discharged horses. Ten owners preferred to answer the same questions in a questionnaire sent by e-mail, and 11 were not reached at all. The first follow-up was conducted during 2010 and the second during 2014. The following information about the horse was recorded: alive/dead; date and reason for death; general owner estimated status (poor/satisfactory/good); estimated convalescence time in months (defined as the time it took to return to athletic function after discharge); SSI (defined as purulent or serous discharge from the laparotomy incision for over a 24 h period); incisional hernia and possible repair (yes/no); estimated size of hernia (in cm); number of postoperative colic episodes (during the first/second/third/fourth/fifth postoperative year or later); re-laparotomy (date/cause/outcome); pre- and postoperative use of the horse (ridden sport horse/hobby horse/harness racing/young or in training/breeding/pasture or company horse/retired or geriatric/did not recover); recovery to the previous or intended use (yes/no); return to or start of sporting activity (yes/no); performance capacity compared with the pre-surgical level (worse/same/better); owner satisfaction, cost of treatment and the total cost (euros) of surgery. For young horses, return to use was considered if the horse achieved the preoperatively expected or intended performance capacity and discipline. Horses aged 15 years or older were defined as geriatric [7]. When the horse had more than one surgery, follow-up data were collected following the last surgery, horses with more than one surgery were included in the analysis once. SSI was defined as a long-term complication and thus it was also included in the owner interview. In all SSI cases, horses were discharged before the infection had resolved. In some patients, SSI developed after the horse had been discharged.

### Data analysis

Median survival time after colic surgery was calculated and survival curves were constructed using the Kaplan–Meier method to estimate the survival function in the whole study population (236 horses). Kaplan–Meier life-table-method with un-stratified log-rank tests combined with un-stratified Cox proportional hazard models were used to compare and investigate postsurgical long-term survival between the following groups: age group of the horse (0–14 years/≥15 years), location of the lesion (small intestine/large intestine), breed (Standardbred/Warmblood/Finnhorse/other horse breed/pony) and enterotomy (yes/no) during the surgery.

The effects of pre-selected explanatory variables on owner estimated “Return to the previous/intended use”, “Incidence of postoperative colic episodes”, and “Death during operation (yes/no)” were analyzed with univariable logistic regression. The explanatory variables were age group; location of the lesion; post-operative colic incidences: yes/no; post-operative hernia: yes/no) for “Return to the previous/intended use”, lesion location and enterotomy during surgery for “Incidence of postoperative colic episodes” and enterotomy for “Death during operation (yes/no)”. Each explanatory variable was analyzed separately. Upon finding few statistically significant results with the univariate analyses, it was decided that multivariate models or other advanced statistics were not indicated.

Mann–Whitney U-tests were used to clarify the association between convalescence time (in months) and selected explanatory variables (age group, location of the lesion, SSI: yes/no and incidence of post-operative colic). Convalescence time was used as the response and explanatory variable as the grouping variable. As the distribution of convalescence time clearly differed from the normal distribution, a non-parametric approach was selected. Owner satisfaction was assessed during the interview and the median owner satisfaction was derived from these subgroups and rounded to the nearest integer. The relationship between the median owner satisfaction and the explanatory variables (hospitalization time in days, convalescence time in months, end sum of the discharge bill, owner estimated return to the same/intended use, postoperative SSI, incidence of post-operative colic) were investigated using cumulative logistic regression separately for each explanatory variable. The analyses were constructed to estimate the probability for higher owner satisfaction values.

In the survival analyses, the results were quantified with Hazard ratios (HR) and their 95% confidence intervals (CI). In the logistic regression and cumulative logit-models the results were quantified with odds ratios (OR) and their 95% confidence intervals. *P* value <0.05 was considered statistically significant. All statistical analyses were performed using SAS® System for Windows, version 9.3 (SAS Institute, Cary, NC, USA).

## Results

### Signalment

Gender, age, breed and preoperative use of the operated horses are presented in Table 1. The mean age of the horses at the time of the surgery was 8.9 years (median 8.5, range 4 days—22.2 years). Warmblood horses were the most prominent breed with 38.6% of the study population. The group ‘Other horse breed’ (n = 28) comprised 9 Icelandic horses, 6 horses of mixed breeds, 3 Irish cobs, 2 Toric horses, 2 Friesians, 2 Shire horses, 1 Estonian native horse, 1 Arabian horse, 1 North Swedish horse and 1 Haflinger. The group ‘Pony’ (n = 12) comprised 5 ponies of mixed breeds, 4 Shetland ponies, 1 Gotland Russ, 1 New Forest and 1 Connemara pony. Approximately two-thirds of the horses (67.4%; 159/236) were insured at the time of the first laparotomy, and 85.8% (12/14) at the time of the re-laparotomy.

**Table 1:** The signalment, short- and long-term outcome and pre- and postoperative use of the operated horses

Signalment	Small intestine (%^a^)	Large intestine (%^a^)	Total (n = 236)
Operated patients in total	72 (30.5%)	164 (69.5%)	236
Gender			
Mare	30 (25.9%)	86 (74.1%)	116 (49.2%)
Gelding	23 (29.1%)	56 (70.9%)	79 (33.5%)
Stallion	19 (46.3%)	22 (53.7%)	41 (17.4%)
Age group (years)			
0–14	58 (29.1%)	141 (70.9%)	199 (84.3%)
Over 15	14 (37.8%)	23 (62.2%)	37 (15.7%)
Breed			
Warm blood	24 (26.4%)	67 (73.6%)	91 (38.6%)
Finnhorse	19 (25.0%)	57 (75.0%)	76 (32.2%)
Standardbred	17 (58.6%)	12 (41.4%)	29 (12.3%)
Other horse breed	7 (25.0%)	21 (75.0%)	28 (11.9%)
Pony	5 (41.7%)	7 (58.3%)	12 (5.1%)

Use of the horse	Preoperative (n = 135)	Postoperative (n = 135)
Ridden sport horse	48 (35.6%)	39 (28.9%)
Hobby horse	46 (34.1%)	54 (40.0%)
Harness racing	19 (14.1%)	20 (14.8%)
Young/in training	13 (9.6%)	1 (0.7%)
Breeding	8 (5.9%)	11 (8.1%)
Pasture/company horse	1 (0.7%)	4 (3.0%)
Retired/geriatric	0 (0.0%)	1 (0.7%)
Did not recover back to use	–	5 (3.7%)

Outcome	Small intestine (n = 72)	Large intestine (n = 164)	Total (n = 236)
Recovered from anesthesia	53 (73.6%)	142 (86.6%)	195 (82.6%)
Euthanasia during operation	19 (26.4%)	22 (13.4%)	41 (17.4%)
Death during hospitalization postop	15 (20.8%)	34 (20.7%)	49 (20.8%)
Discharged from hospital	38 (52.8%)	108 (65.9%)	146 (61.9%)
No information, long-term follow-up^b^	2 (2.8%)	6 (3.7%)	8 (3.4%)
No information after discharge^c^	1 (1.4%)	2 (1.2%)	3 (1.3%)

Horses (n = 236) operated for colic between 2006 and 2012 in University of Helsinki, Equine Teaching Hospital, Finland

^a^Percentages calculated out of the values in the Total-column

^b^Horses whose owners could not be reached and therefore specific data on postsurgical convalescence could not be acquired. These horses were included in the survival analysis as the date and reason of death were available from the hospital records and national databases (Heppa® database of the Finnish Trotting Association Hippos, Helsinki, Finland)

^c^Horses of which no records and information were available after discharge. These horses were excluded from all the statistical analyses

### Surgery and short-term survival

Survival from surgery and outcome of the operated horses is presented in Table 1. In total 17.4% (41/236) of the operated horses were euthanized during surgery, 82.6% (195/236) recovered from anesthesia, and subsequently 20.8% (49/236) were euthanized during hospitalization after the surgery and 74.9% (146/195) of the horses that survived the surgery were discharged. Diagnosis categories, surgical findings, procedures and postsurgical complications are presented in Table 2. Most common diagnosis during the surgery were non-strangulating displacement of the large intestine (37.3%; 88/236), strangulation of small intestine (22.0%; 52/236) and strangulating displacement of large intestine (20.3%; 48/236). Five of the operated patients had a gastric rupture or bowel perforation with no other findings. Enterotomy was performed in 50.4% of the operated patients. Postsurgical reflux (defined as reflux of more than 2 l) was the most common complication immediately after surgery and it affected 27.2% of the operated patients. The effect of enterotomy during surgery and lesion location on discharge rate, incidence of postoperative colic and the probability of returning to the same use are presented in Table 3. A second laparotomy was performed in 5.9% (14/195) of the operated patients, 11 of which occurred during the immediate postoperative period and 3 were performed later after discharge due to postoperative colic. Two horses had to be euthanized during the second surgery. The mean hospitalization time after surgery was 7.2 days (median 6.5 days, range 0–31 days); for horses with small intestinal lesion 6.0 d (median 5.0, range 0–21 days) and large intestinal lesion 7.7 days (median 7.0, range 0–31 days), respectively.

**Table 2:** Categorised diagnosis, surgical findings, procedures and complications of the operated horses

Diagnosis category (n = 236)	Total	%
Small intestine		
Strangulating displacement	52	22.0
Simple obstruction	8	3.4
Large intestine		
Non-strangulating displacement and obstruction	55	23.3
Non-strangulating displacement	33	14.0
Strangulating displacement and obstruction	27	11.4
Simple obstruction	26	11.0
Strangulating displacement	21	8.9
Other		
Anterior enteritis	5	2.1
Gastric/bowel perforation with no other findings	5	2.1
Primary gas accumulation	4	1.7

Surgical findings^a^ (n = 236)	Small intestine	Caecum	Colon	Small colon	Total
Displacement of large intestine	–	–	136	–	136 (57.6%)
Right dorsal displacement	–	–	49	–	49
Other displacement			36		36
Flexion, large intestine	–	–	27	–	27
Left dorsal displacement	–	–	24	–	24
Intestinal herniation	32	–	–	–	32 (13.6%)
Foramen epiploicum	13	–	–	–	13
Inguinal	10	–	–	–	10
Other	9	–	–	–	9
Sand accumulations	–	–	28	–	28 (11.9%)
Mesenterial anomaly	17	–	1	–	18 (7.6%)
Ruptured bowel	–	–	11	–	11 (4.7%)
Lipoma	10	–	–	–	10 (4.2%)
Foreign objects	–	–	–	3	3 (1.3%)
Adhesions	1	–	1	–	2 (0.8%)
Roundworm	1	–	–	–	1 (0.4%)

Surgical procedures^a^ (n = 236)	Small intestine	Caecum	Colon	Small colon	Total
Enterotomy	2	–	117	–	119 (50.4%)
Gas removal^b^	NI	NI	NI	NI	95 (40.3%)
Resection	17	–	1	–	18 (7.6%)

Postsurgical complications	Total	%
Short-term (n = 195)		
Reflux/ileus	53	27.2
Peritonitis	14	7.2
Re-laparotomy^c^	11	5.6
Incisional dehiscence	4	2.1
Laminitis	3	1.5
Cardiac failure	2	1.0
Liver failure and coagulopathy	1	0.5
Long-term: (n = 135)		
Colic episodes in 8y10mo	52	38.5
Colic in the first year postop	27	20.0
Only one colic episode	21	15.6
Re-laparotomy due to colic	3	2.2
Colic episodes/horse year	0.18	
Surgical site infection SSI	39	28.9
Incisional hernia	15	11.1

Horses (n = 236) operated for colic between 2006 and 2012 in University of Helsinki, Equine Teaching Hospital, Finland

^a^ One horse may have had more than one finding or procedure, therefore the listed findings and procedures do not exclude each other

^b^ Segment of intestine: *NI* no information

^c^ Re-laparotomy during hospitalization due to continuous pain, colic, reflux, or incisional dehiscence

**Table 3:** The effect and significance of different variables on outcome parameters

Response	Explanatory variable	*P* value	Odds ratio	95% CI^a^
Discharge from the hospital	Enterotomy (no vs. yes)	0.09	0.63	0.37; 1.07
Postoperative colic	Enterotomy (no vs. yes)	0.31	0.69	0.34; 1.40
	Lesion location (LI vs. SI^b^)	0.01	3.27	1.31; 8.19
Return to the same/intended use	Age group (0–14 vs. ≥15)	0.88	1.10	0.33; 3.62
	Postoperative colic (no vs. yes)	0.47	1.41	0.56; 3.54
	Hernia (no vs. yes)	0.74	0.77	0.16: 3.68
	Lesion location (LI vs. SI^b^)	0.49	1.42	0.53: 3.83

^a^ 95% CI 95% confidence interval

^b^ Large intestinal vs. small intestinal lesion

### Patterns of survival

**Fig. 1:**
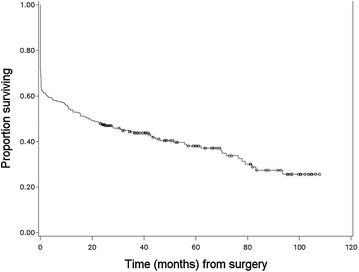
Kaplan–Meier-plot of the overall survival rate following colic surgery. Number of operated horses 236, time 0 = time of surgery

**Fig. 2:**
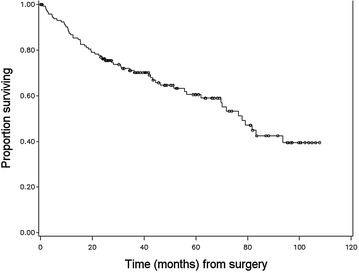
Kaplan–Meier-plot of the overall survival rate following colic surgery. Number of discharged horses 143, time 0 = discharge from hospital

**Fig. 3:**
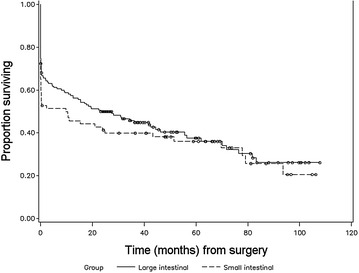
Kaplan–Meier-plot of overall survival between small and large intestinal lesions. Number of operated horses 236, time 0 = date of surgery

**Fig. 4:**
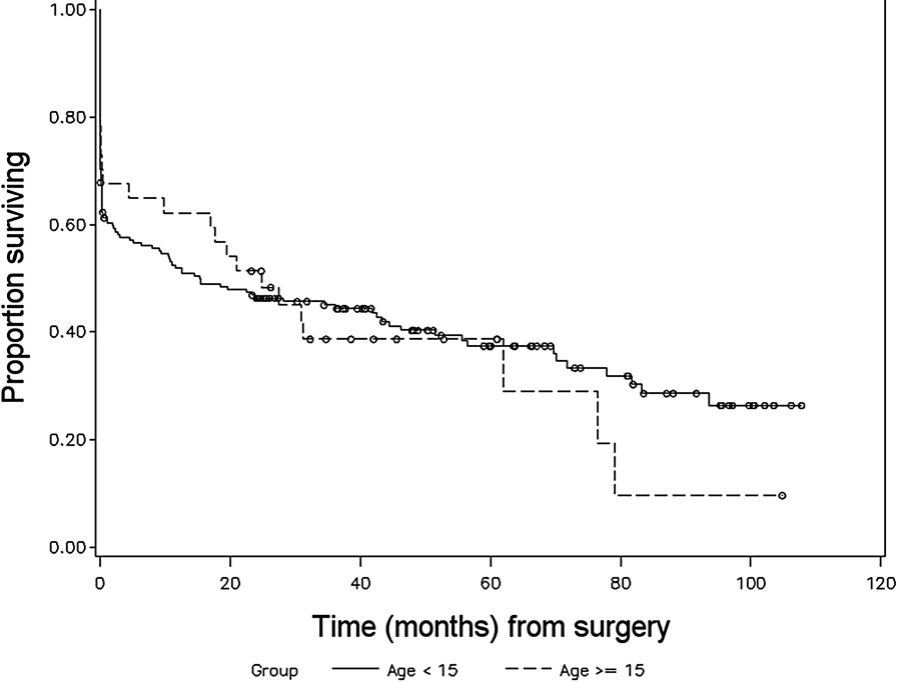
Kaplan–Meier-plot of overall survival rate between age groups. Number of operated horses 236, time 0 = date of surgery. Horses between 0 and 14 years and horses ≥15 years

Figures 1, 2, 3 and 4 illustrate the patterns of survival for the operated colic horses. A marked mortality can be seen immediately following surgery in Fig. 1. Figure 2 illustrates the overall survival of the discharged horses. After 5 years, the cumulative survival was approximately 0.6 and 0.4 after 8 years and 10 months when the follow-up study was ended. Figure 3 illustrates the survival between small and large intestinal lesion groups. Mortality was higher in horses with small intestinal lesions up till approximately 40 months after surgery when cumulative survival was 0.4. The survival was similar between the two groups from 3 to 7 years. Figure 4 illustrates the pattern of overall survival of the different age groups of horses. Geriatric horses (≥15 years) had a less steep curve initially but from 30 to 70 months the curves of both age groups had the same gradient. The cumulative survival of the geriatric horses from 6 to 9 years after surgery was 0.1.

### Long-term convalescence

Survival information (date and reason of death) was obtained from 97.9% (143/146) of the discharged horses. We did not obtain follow-up information after discharge for eight of the discharged horses. These eight horses were, therefore, excluded from the rest of the study except for the survival analysis. Full follow-up data (information including convalescence after discharge) were acquired in 92.5% (135/146) horses and completed at 8 years and 10 months. The median survival time for the discharged 143 horses was 79.2 months (Fig. 2). The survival rates at 6, 12, 24, 36, 48 and 60+ months were as follows: 90.2% (129/143), 83.9% (120/143), 73.4% (105/143), 52.4% (75/143), 37.1% (53/143) and 28.7% (41/143). Age group (0–14 years/≥15 years) or location of the lesion (small intestinal/large intestinal) had no significant effect on the overall probability for survival of the patient after colic surgery long-term.

The mean owner-reported convalescence time of the horse was 6.0 months (median 6.0, range 0–20 months). A majority of the owners (87.4%; 118/135) reported the horse to be in good or satisfactory condition after this period. The effects of age group, lesion location, postoperative colic and SSI variables on the convalescence time are presented in Table 4. Horses with a small intestinal lesion tended to have a slightly longer time postoperatively to return to expected athletic function compared to the horses with large intestinal lesion (Table 4, *P* = 0.06). SSI seemed to lengthen the convalescence time but this increase was not statistically different (Table 4).

**Table 4:** The association and significance of explanatory variables on the convalescence time

Effect of the parameter on the convalescence time (months)	Z value	*P* value
Age group (0–14 vs. ≥15)	−0.59	0.55
Lesion location (LI vs. SI^a^)	1.90	0.057
Postoperative colic (no vs. yes)	−1.42	0.15
Wound infection/SSI (no vs. yes)	0.99	0.32

^a^ Large intestinal vs. small intestinal lesion

### Long-term complications

Long-term complications and different variables that affect them are presented in Tables 2 and 3. During the first postoperative year 20.0% (27/135) of the horses suffered from one or more colic episodes. Postoperative colic was documented in 38.5% (52/135) of the discharged horses during the long-term surveillance. The incidence of colic in the operated horses was 0.18 per horse year. The horses with a large intestinal lesion were 3.3-fold more prone to have postoperative colic episodes (CI 1.3; 8.2, *P* = 0.01) compared with the horses with small intestinal lesion. Three horses (2.2%; 3/135) had to be operated after discharge because of colic. Surgical site infection, defined as incisional drainage, was evident in 28.9% (39/135) cases and 11.0% (15/135) of the discharged horses developed an incisional hernia at some point postoperatively.

### Postoperative performance

Pre- and postoperative use of the horses is presented in Table 1. The majority of the operated horses (83.7%; 113/135) were estimated to have reached previous or intended use after the colic surgery, 78.5% (106/135) reached their preoperative or better level of performance. Owners estimated that 72.8% (59/81) of the horses which had been used in previous competitions had also competed at least once postoperatively during the study period and that 89.8% (53/59) performed on the same or at an better level than preoperatively. When the official national database records were searched, 48.9% (66/135) preoperatively and 35.6% (48/135) postoperatively of the discharged horses had competed in one or more discipline. The effects of different variables on the probability of returning to the same/intended use are presented in Table 3. None of the variables were found to have had a significant effect on the patient’s probability of regaining previous or intended level of performance.

### Owner satisfaction

Nearly all, 96.3% of the owners graded the veterinary care and 98.5% rated their satisfaction toward colic surgery to range from satisfactory to good. Different variables (the length of hospitalization time; number of postoperative colic episodes; SSI; the length of the convalescence time in months; whether or not the horse returned to the same use; the sum of the total cost of veterinary care) did not have a significant negative effect on the median owner satisfaction level.

## Discussion

This study is a retrospective follow-up on the long-term survival up to 8 years and 10 months after colic surgery. Long-term retrospective studies are challenging as from 14 to 44% of the horses are lost to follow-up [2, 7, 14–16]. Missing data and small study population sizes may cause bias in statistical analysis and interpretation of results. Information on survival in this study was obtained for nearly all of the discharged horses (97.9%; 143/146) and full follow-up data with complications and their effect on the use and postoperative performance were obtained for 92.5% (135/146) of the discharged horses.

Short-term survival rate after colic surgery was somewhat lower (82.6%; 195/236) compared with previous studies (85.7–87.0%) [11, 17, 18]. Two-thirds (67.4%; 159/236) of the horses were insured at the time of the first surgery, and (85.7%; 12/14) at the time of the second surgery. Most insurance companies in Finland will not pay out the life insurance compensation sum unless the indicated and possible abdominal surgery has actually been attempted. Therefore, the insurance status may have necessitated horses with poor prognosis to be operated on and then euthanized during surgery or shortly afterwards. Additionally, there are only two 24-h equine emergency hospitals in the southern part of Finland. This might cause delays in the initiation of the treatment, which worsens short- and long-term prognosis for colic patients [14, 16, 17, 19].

The incidence of postoperative colic during the first year after the surgery was 20.0% (27/135) and for the whole study period the incidence was 0.18 colic cases per horse year. Postoperative colic is reported by several other studies to be the most common long-term complication after colic surgery, documented in between 11 and 35% of the patients, which is a negative factor for postoperative survival [1–4, 14, 17, 19, 20]. A study by Tinker and colleagues [21] reported the incidence of colic in a large population of horses that had not had colic surgery, to vary between 0 and 30%. The crude incidence density rate of colic cases has been estimated as 0.106 colic cases per horse year [21]. Horses in this study had postoperatively 1.7 times higher incidence of colic than the naive horse population with no history of colic surgery that was reported by Tinker and colleagues [21]. Previous reports found that operated horses have had 2.8–7.6 times higher incidences of colic compared to non-operated horses [1].

The number of postoperative colic episodes in this study was estimated subjectively by the owner. Not all episodes required veterinary treatment. Therefore, the capability of owners to recognize colic can be questioned and this circumstance may introduce some bias in the results. However, postoperative colic in this study was found to be 3.3-fold more common in horses that had been operated because of a large intestinal lesion compared with the small intestinal lesion. A total of 28 horses in the present study had large sand accumulations in the large colon. Previous reports of sand accumulations in the large intestine were found to be a common cause of colic and abdominal discomfort with the Finnish horse population [22]. If the management of the horse is not changed or the risk factors are not removed, then the horse can be more prone to suffer repeated colic episodes later in life. Enterotomy due to secondary impaction, was performed in the majority of the operated cases in the pelvic flexure in the present study. However, it did not statistically increase the incidence of postoperative colic. Therefore, the increased risk of postoperative colic cannot solely be explained with the existence of possible peritonitis and adhesion formation subsequent to enterotomy. During the immediate postoperative period, 5.6% (11/195) of the patients underwent a second laparotomy due to recurrent colic, ileus or incisional dehiscence.

Re-laparotomies were performed in 7.2% (14/195) of the horses; 11 during the immediate post-operative period and 3 later on after discharge. The number of re-laparotomies has been higher in other studies (9.6–20.0%) [1, 11, 22–25]. The financial situation of the owners and the use of horses most likely played a role in deciding on re-operating, as most of the horses in this study were hobby horses. It should be noted, however, that a majority of re-operated patients in this study were insured and it is assumed that the insurance status played an important role when deciding whether to re-operate. Owners with no insurance who had instead decided to have their horses euthanatized than to be subjected to a second colic surgery most likely did so due to financial limitations.

Surgical site infection was recorded both during hospitalization and also in the owner questionnaire in this study. SSI was categorized as a long-term complication, as in all cases the horses were discharged before the infection had resolved. In some cases, infection became evident only after discharge. The occurrence of SSI often leads to incisional hernia [2] and incisional hernia in this study developed in 11.1% (15/135) of the cases. A total of 3 hernias had to be corrected surgically, the size of the deficit being approximately 10, 30 and 35 cm, respectively. Hernias are usually identified 2–12 weeks postoperatively [14]. The extended follow-up enabled us to document incisional complications that had also developed later after discharge. Interestingly, the development of hernia did not have a negative effect on the owner-reported return to use and performance level attained for these horses. In other studies, SSI has been reported in 11–42% [1, 14, 19] and incisional hernia in 6–17% of horses following laparotomy [6, 7, 14]. Horses that develop an incisional hernia have been reported to have a poorer outcome and be 7–14 times less likely to return to sporting activity or athletic use [6, 7]. However, in previous studies, the number of horses with incisional hernia is limited: 12/195 horses in one study [7]. The number of horses with incisional hernia was not actually reported in the other study of 79 horses and the ORs those authors calculated were also associated with wide confidence intervals [6]. Limited owner finances, which prevented hernia repair, may have also encouraged the owners to attempt to train and use the horses for the intended activity and performance. In our experience, a small abdominal wall defect does not incapacitate the horse from athletic activity.

If the horse survived to discharge, the overall estimated median survival time was 79.2 months (Fig. 2). Survival at 12 months (83.9%; 120/143) is comparable with an earlier report from Denmark by Christophersen and colleagues (86.6%) [6]. The overall survival between their study and our present data are very similar up to 36 months after surgery. The horses in our study were older (mean 9.2 years vs. 6 years), the population was also larger (143 vs. 79 horses) and the follow-up time was longer (8 years 10 months vs. 5 years) [6]. The decline in survival rate from 5 years onwards probably follows the life expectancy of horses. As expected, survival of horses operated for small intestinal (Fig. 3) lesion was somewhat poorer compared to horses operated for large intestinal lesion in long-term follow-up till about 36–40 months after surgery. Interestingly, after 40 months, the survival graphs were similar between the large and small intestinal groups, till about 90+ months. Complications are common during and after small intestinal surgery, and according to other reports these horses tend to have slightly poorer postoperative prognosis [1, 2, 11, 14, 22, 26]. However, this study supports the finding that a number of horses operated for small intestinal colic are able to live and perform surprisingly long, even up to 10 years after surgery [14]. The age of the horse (0–14 years/≥15 years) did not have a significant effect on overall survival (Fig. 4). Similar findings have been reported in the short-term follow-up in two previous studies [4, 27] but long-term follow-up studies are missing. The number of aged horses was small in this study, 16% of the population.

A high prevalence of discharged horses in the present study returned to the intended use (83.7%; 113/135) and the horses were also able to perform on the same or even at a better level (78.5%; 106/135) after the colic surgery. At the time of surgery, 13 horses were young or were in training and by the time of the postoperative interview 12 of these horses had already moved into a particular discipline and were performing in their intended use. Athletic performance was subjectively determined by the horses’ owners, they can be considered to be reliable in assessing performance levels and/or changes within a certain time frame. Nevertheless, a subjective opinion cannot be compared to numerical objective data, such as racing earnings or the number of starts, which reflect the measurable results and changes in performance. It should be noted that most of our horses were, however, hobby horses and therefore could not be categorized or analyzed using only information on competition records. Nevertheless, the information on prognosis, use and capacity to perform is also important to the owners.

We also studied the official competition record databases, which support this as 72.8% (59/81) of horses with a competition history had officially competed postoperatively. The difference between the owner estimation and actual national database records can be explained by the fact that smaller competitions are not included in the national databases. Only a few studies about the long-term performance ability and competing after colic surgery in a population of horses of different breeds exist [6, 7]. Moreover, differences in the study populations and use of the horses between the few studies that do exist may affect the results. A recently published paper studied the operated horses surviving past 6 months after discharge. As much as 86.1% (68/79) of the horses with sporting activity before surgery had resumed by 6 months post-operation and 83.5% (66/79) reached their pre-surgical level of performance [6]. Another report documented that at 12 months after discharge, 76% (145/190) of the horses were performing in their intended use, and 66% (101/153) were doing so at the previous or better level of performance activity [7]. Thoroughbreds have been studied as racing or competitive horses but they are often retired early from their career [11–13]. Two studies on racing Thoroughbreds, respectively reported majorities of 69.0% (59/85) and 76.0% (45/59) that had returned to racing at least once after colic surgery with no marked changes in performance levels [12, 13]. Differences in study populations and the use of the horses most likely affects the results to some extent and therefore the results in their present form are not entirely comparable.

## Conclusions

The findings of the present study suggest that horses with small intestinal lesions tended to have a longer convalescence time than horses with large intestinal lesions and that the incidence of postoperative colic was relatively low compared with earlier reports. However, horses operated because of large intestinal lesion were 3.3 times more prone to have postoperative colic compared with horses with small intestinal lesions. Additionally incisional hernia and incidence of postoperative colic—in contrast to previous studies—as well as the age of the horse, location of the surgical lesion and convalescence time, did not decrease the probability of performance after the surgery. Most importantly, the majority of discharged horses had a good prognosis for long-term survival, were able to return to their intended use, compete postoperatively on a satisfactory level and also perform for several years after the operation. A majority of owners estimated that the veterinary care and general satisfaction to be satisfactory or above and also expressed their general satisfaction.
